# Mortality and causes of death among patients with opioid use disorder receiving opioid agonist treatment: a national register study

**DOI:** 10.1186/s12913-019-4282-z

**Published:** 2019-07-02

**Authors:** Anne Berit Bech, Thomas Clausen, Helge Waal, Jūratė Šaltytė Benth, Ivar Skeie

**Affiliations:** 1 National Advisory Unit on Concurrent Substance Abuse and Mental Health Disorders, Innlandet Hospital Trust, Department of Mental Health, P.O. Box 104, N-2381 Brumunddal, Norway; 2 Norwegian Centre for Addiction Research (SERAF), Institute of Clinical Medicine, Oslo University, P.O. Box 1171, Blindern, N-0318 Oslo, Norway; 3 National Advisory Unit on Substance Use Disorder Treatment, Oslo University Hospital, Sognsvannsveien 21, Bygg 6, P.O. Box 4959 Nydalen, N-0424 Oslo, Norway; 4 Institute of Clinical Medicine, Campus Ahus, Oslo University, P.O. Box 1171, Blindern, N-0318 Oslo, Norway; 5 Health Services Research Unit, Akershus University Hospital, P.O. Box 1000, N-1478 Lørenskog, Norway; 6 Regional Psychiatric Centre Gjøvik, Innlandet Hospital Trust, Kyrre Grepps gate 11, N-2819 Gjøvik, Norway

**Keywords:** Opioid agonist treatment, Mortality, Cause of death, Multimorbidity, Overdose, Methadone, Buprenorphine

## Abstract

**Background:**

Mortality rates and causes of death among individuals in opioid agonist treatment (OAT) vary according to several factors such as geographical region, age, gender, subpopulations, drug culture and OAT status. Patients in OAT are ageing due to effective OAT as well as demographic changes, which has implications for morbidity and mortality. Norway has one of the oldest OAT populations in Europe. Because of the varying mortality rates and causes of death in different subgroups and countries, research gaps still exist. The aims of this study were to describe the causes of death among OAT patients in Norway, to estimate all-cause and cause-specific crude mortality rates (CMRs) during OAT and to explore characteristics associated with drug-induced cause of death compared with other causes of death during OAT.

**Methods:**

This was a national, observational register study. Data from the Norwegian Cause of Death Registry and the Norwegian Patient Registry were combined with data from medical records. We included all patients in the Norwegian OAT programme who died not more than 5 days after the last intake of OAT medication, between 1 January 2014 and 31 December 2015.

**Results:**

In the 2-year observation period, 200 (1.4%) of the OAT patients died. A forensic or medical autopsy was performed in 63% of the cases. The mean age at the time of death was 48.9 years (standard deviation 8.4), and 74% were men. Somatic disease was the most common cause of death (45%), followed by drug-induced death (42%), and violent death (12%). In general, CMRs increased with age, and they were higher in men and in patients taking methadone compared with buprenorphine. Increasing somatic comorbidity, measured by the Charlson comorbidity index, reduced the odds of dying of a drug-induced cause of death compared with other causes of death.

**Conclusions:**

Both somatic and drug-induced causes of death were common during OAT. Improved treatment and follow-up of chronic diseases, especially in patients aged > 40 years, and continuous measures to reduce drug-induced deaths appear to be essential to reduce future morbidity and mortality burdens in this population.

## Background

Opioid use disorder (OUD) is associated with high rates of morbidity and mortality [1]. Individuals who use illicit opioids have up to 15 times the risk of premature mortality compared with the general population [2]. Opioid agonist treatment (OAT) with methadone, buprenorphine or buprenorphine-naloxone is the most common evidence-based treatment modality for individuals with OUD. It is well established that OAT substantially reduces mortality, especially overdose deaths [1–4]. Criminal convictions and somatic morbidity related to substance use and drug injection (e.g., local and systemic bacterial infections) are also reduced during OAT, and quality of life is improved [1, 5–7].

Mortality rates and causes of death among individuals in OAT vary according to factors such as geographical region, age, gender, subpopulations, cohort characteristics, drug culture (i.e., injection), retention in treatment and OAT status [2, 4]. A systematic review and meta-analysis published in 2018 found a pooled all-cause crude mortality rate (CMR) of 0.93 per 100 person-years (PY) (95% confidence interval [CI]: 0.79–1.04) during OAT compared with 4.89/100 PY (CI 3.54–6.23) for untreated periods and 1.69/100 PY (CI 1.47–1.91) after cessation of OAT [4]. In general, CMRs increase with age, especially for somatic causes of death [8], and men have higher CMRs compared with women [2, 4]. CMRs also appear to be higher for individuals taking methadone compared with those taking buprenorphine during induction and treatment, and after cessation of OAT [2–4]. However, retention in treatment is better with methadone [9], and retention in OAT for more than 1 year is associated with a lower mortality rate [4].

OAT patients in Europe are ageing due to effective OAT as well as demographic changes as the post-war baby boom generation ages [10]. This ageing trend can also be seen in Australia and in the USA [10, 11], and has implications for morbidity and mortality. Norway has one of the oldest OAT populations in Europe [12], with a mean age of 44.9 years in 2017 [13]. As OAT patients are getting older, somatic causes of death will likely increase [14], although high drug-induced mortality, irrespective of gender, has been found among ageing methadone patients in recent studies from Scotland and England, including high methadone-specific mortality rates in patients aged > 45 years [15, 16].

Research gaps still exist because of varying mortality rates and causes of death in different subgroups and countries. In Norway, mortality data on individuals receiving OAT are more than 10 years old [8, 17], and no recent studies have linked data to the mortality register [14], which is essential to obtain reliable data about the causes of death. To improve treatment and prevent premature mortality, more research is warranted to better understand mortality rates and the distribution of causes of death in an ageing OAT population. The aims of this study were to describe the causes of death among OAT patients in Norway, to estimate all-cause and cause-specific CMRs during OAT in patients stratified by age, OAT medication and gender, and to explore characteristics associated with drug-induced cause of death compared with other causes of death during OAT.

## Methods

### Study design and setting

This study was a national, observational study combining register and hospital record data. In Norway, OAT is delivered within a national OAT programme and encompasses both abstinence-oriented treatment and harm-reduction goals. Addiction units in the specialist health care system assess the need for OAT and initiate treatment, and the treatment involves collaboration between addiction units, general practitioners (GPs) and health and social services in the municipalities [18, 19]. At the end of 2017, 7622 patients were enrolled in OAT, 38% of whom used methadone and 59% used buprenorphine or buprenorphine-naloxone [13].

We included all patients in the national OAT programme who died between 1 January 2014 and 31 December 2015. According to the national guidelines, patients who have missed doses for more than 5 consecutive days must be restarted on OAT medication because of potential loss of opioid tolerance. Thus, patients were included if they had died during ongoing treatment or not more than 5 days after the last reported intake of OAT medication. Clinicians in addiction units initially reported 255 deceased OAT patients. Fifty-five patients were excluded: 44 patients who died more than 5 days after the last reported intake of OAT medication, eight patients whom the hospitals were unable to identify further and three patients whose OAT status was unknown at the time of death. A total of 200 patients met the inclusion criteria.

### Measurements

Data from the Norwegian Cause of Death Registry and the Norwegian Patient Registry (NPR) were combined with hospital record data. Clinicians completed a questionnaire about the patient’s age, gender, health region, OAT medication at the time of death, duration of OAT treatment and information about prescription drugs used before death. At least one prescription of benzodiazepines (BZD) or z-hypnotics during the year before death registered either in the questionnaire or in the medical record was dichotomized into one variable called “BZD/z-hypnotic medication”. At least one prescription of antidepressants and/or antipsychotic drugs was dichotomized into one variable called “Psychotropic medication”. From the hospital records, we also collected the OAT status report for the year of death and 3 years before death, if available. The OAT status report is an annual individual report on all OAT patients and is based on the clinician’s knowledge of the patient’s situation; this report is preferably completed in collaboration with the patient. The variables “Disability/retirement pension”, “Own home” and “OAT prescribed by GPs” were collected from the OAT status reports.

Data on the cause of death, place of death, main intoxicant in drug-induced deaths and whether the deceased had an autopsy or not were obtained from the Norwegian Cause of Death Registry [20]. The underlying cause of death is defined as “the illness or injury which initiated the train of morbid events leading directly to death or the circumstances of the accident or violence which produced the fatal injury” [21]. The underlying cause of death was categorized into one of three main groups: death due to somatic disease, drug-induced death and violent death. The definition of drug-induced death is based on the International Classification of Diseases, 10th edition (ICD-10) and includes unintentional overdose or overdose by unknown intent, intentional overdose and substance use disorder [21, 22]. Violent deaths include deaths due to accident, suicide (except intentional overdose) and homicide.

The NPR contains information about all patients referred to or having received treatment in the specialist health care service in Norway [23]. From the NPR, we collected information on admissions to psychiatric hospitals and the diagnoses based on the ICD-10 in the 5 years before death. For each patient, we derived a Charlson comorbidity index score, which is a widely used measure of disease burden based on age and ICD-10 diagnoses for 17 somatic conditions [21, 24, 25]. The variable “Previous non-fatal overdose(s)” refers to either non-fatal overdoses registered in the OAT status report or hospital admission due to non-fatal intoxication (ICD-10 codes T4n, T50.9 and T56.9) registered in the NPR in the 5 years before death.

Data were collected in 2017 and 2018; however, to minimize recall bias, the questionnaire used in the study was filled out by the clinicians shortly after the patients had died in 2014 and 2015. In six cases, the cause of death was not registered or was unknown in the Cause of Death Registry but was found in the medical records. Thus, the cause of death could not be established in only two of 200 patients.

### Statistical analysis

The characteristics of all patients and stratified by causes of death were described by frequencies and percentages if categorical, and means and standard deviations (SD) or medians and minimum and maximum values if continuous. Group comparisons were made using Student’s t test or Mann–Whitney U test for continuous data and χ^2^ or Fisher’s exact test for categorical data. To obtain more balanced CMRs, data for the years 2014 and 2015 were combined due to the small number of expected deaths. The CMRs were calculated by dividing the total number of deaths in OAT by the total number of patients in OAT mid-year 2014 and 2015 (i.e., PY), for all patients as well as stratified by age, OAT medication and gender. CMRs are reported per 100 PY, with 95% Poisson CIs [26]. Bivariate and multiple multilevel regression models were estimated to assess the association between drug-induced cause of death and patient characteristics included as fixed effects into the models. Random intercepts for region were included to correctly adjust the estimates for within-region correlations. The results are presented as odd ratios (ORs) and 95% CIs, with other causes of death used as reference category. The regression models were estimated on cases with no missing values of covariates. The results with *p* < 0.05 were considered statistically significant, and all tests were two-sided. The analyses were performed using IBM SPSS Statistics for Windows version 25 (IBM Corp.), Stata Statistical Software version 15 (StataCorp LLC) and SAS version 9.4.

### Ethical considerations

The study was approved by the Regional Committee for Medical and Health Research Ethics South-East (Case number 2016/1204), the Cause of Death Registry, the NPR and the participating hospital trusts, including data protection officials.

## Results

### Patient characteristics

Table 1 gives an overview of the patient characteristics. The mean age at the time of death was 48.9 years (SD 8.4, ranging from 23 to 71 years), and 74% (*n* = 147) were men. Methadone was used by 55% of patients, at a median dose of 90 mg (ranging from 15 to 200 mg), and buprenorphine was used by 41% at a median dose of 16 mg (ranging from 1 to 52 mg). GPs prescribed OAT medication for 68% (*n* = 156) of the patients. The median total duration of OAT was 8 years (ranging from 1 month to 17 years). Four patients had been in OAT for < 3 months at the time of death.

**Table 1:** Characteristics of 200 patients who died during opioid agonist treatment, stratified by the cause of death

Variables	N	Total, N (%)	Drug-induced deaths	All other causes of death
			*N* = 84, n (%)	*N* = 116, n (%)
*Demographics*				
Male gender	200	147 (74)	59 (70)	88 (76)
Age, mean (SD)	200	48.9 (8.4)	46.9 (8.5)	50.3 (8.2)
Region East,	200	89 (45)	30 (36)	59 (51)
incl. Capital Oslo				
Region South	200	39 (20)	16 (19)	23 (41)
Region West	200	44 (22)	26 (31)	18 (16)
Region Mid-Norway	200	14 (7)	7 (8)	7 (6)
Region North	200	14 (7)	5 (6)	9 (8)
Disability/retirement pension^a^	154	117 (76)	45 (70)	72 (80)
Own home^b^	162	125 (77)	54 (78)	71 (76)
*OAT medication*				
Methadone	199	109 (55)	46 (55)	63 (54)
Buprenorphine	199	82 (41)	35 (42)	47 (41)
Other	199	8 (4)	2 (2)	6 (5)
OAT prescribed by GPs	156	106 (68)	42 (60)	64 (74)
*Dose methadone (met) or buprenorphine (bup)*				
< 60 mg met or < 8 mg bup	187	21 (11)	10 (12)	11 (10)
60–120 mg met or 8–24 mg bup	187	141 (75)	62 (77)	79 (75)
> 120 mg met or > 24 mg bup	187	25 (13)	9 (11)	16 (15)
*Total duration of OAT*				
< 4 years	188	36 (19)	14 (18)	22 (20)
4–8 years	188	56 (30)	30 (38)	26 (24)
8–12 years	188	49 (26)	18 (23)	31 (29)
12–17 years	188	47 (25)	18 (23)	29 (27)
*Comorbidities*				
Charlson index score,	200	2.0 (0–12)	1.0 (0–9)	3.0 (0–12)
median (min–max)				
Psychiatric admissions^c^	200	56 (28)	26 (31)	30 (26)
BZD/Z-hypnotics^d^	177	76 (43)	28 (38)	48 (46)
Psychotropic medication^e^	156	44 (28)	12 (20)	32 (33)
Previous non-fatal overdose^f^	200	59 (30)	30 (36)	29 (25)

^a^Only four patients had a retirement pension at the time of death (> 67 years). Among those who did not have a disability or retirement pension, two had paid work and the rest had work assessment allowance or social welfare

^b^Own home, rented or owned. Among those who did not have an own home, two were homeless; the rest lived in shelters, institutions, with friends/family or were in prison

^c^Psychiatric admissions registered in the NPR in the last 5 years before death

^d^BZD/Z-hypnotics prescribed at least once in the year before death

^e^Antidepressants/antipsychotic medication prescribed at least once in the year before death

^f^Non-fatal overdoses registered in the NPR or in OAT status reports in the last 5 years before death

Comorbid conditions were common, as reflected by a median Charlson comorbidity index score of 2. Only 18% of the patients had a Charlson comorbidity index of zero, which corresponds to no registered somatic medical condition in the NPR and aged < 50 years at the time of death. The most frequent chronic diseases registered in the NPR in the 5 years before death were liver diseases (62%, chiefly hepatitis C), cardio-vascular diseases (19%) and chronic obstructive pulmonary disease (COPD) (19%). Co-prescription was common, and 43% of the deceased had at least one prescription of BZD/z-hypnotics in the year before death, and 28% were prescribed other psychotropic medication. Thirty per cent of the patients had experienced previous non-fatal overdose(s) in the last 5 years before death.

Compared with patients taking buprenorphine, patients taking methadone were significantly more likely to live in Health Region East than the other four health regions (75% vs. 61%/31%/29%/46%; all *p* < 0.01) and they had been significantly longer in OAT (median 10.1 vs. 6.8 years; *p* < 0.001), but were not significantly older (mean 49.3 vs. 48.1 years; *p* = 0.331) (data not shown in Table 1).

### Causes of death

Table 2 provides an overview of the causes of death for all patients as well as stratified by gender; 90 deaths (45%) were caused by somatic disease, 84 (42%) were drug induced, and 23 (12%) were violent deaths.

**Table 2:** Causes of death among 200 patients in opioid agonist treatment in Norway, stratified by gender

	Total	Men	Women
	*N* = 200, n (%)	*N* = 147, n (%)	*N* = 53, n (%)
Somatic cause of death	90 (45)	69 (47)	21 (40)
Cancer, excl. Liver cancer	26 (29)	19 (28)	7 (33)
Cardio-vascular disease	20 (22)	15 (22)	5 (24)
Pulmonary disease	18 (20)	14 (20)	4 (19)
Liver disease, incl. Liver cancer	14 (16)	12 (17)	2 (10)
Other somatic cause of death	12 (13)	9 (13)	3 (14)
Drug-induced cause of death^a^	84 (42)	59 (40)	25 (47)
Methadone	31 (37)	21 (36)	10 (40)
Heroin	17 (20)	14 (24)	3 (12)
Other opioids (T402, T404, T406)	15 (18)	10 (17)	5 (20)
Substance use disorder (F11, F19)	17 (20)	11 (19)	6 (24)
Non-opioid overdose	4 (5)	3 (5)	1 (4)
Violent cause of death	23 (12)	16 (11)	7 (13)
Suicide	12 (52)	7 (44)	5 (71)
Accident	8 (35)	6 (38)	2 (29)
Homicide	3 (13)	3 (19)	0
Other/unknown cause of death^b^	3 (2)	3 (2)	0

Data are expressed as *n (%)*. The distributions of somatic cause of death, drug-induced, violent and unknown cause of death did not differ between men and women (*p* = 0.610^a^)

^a^Five suicides by intentional overdose are included in the group “Drug-induced death”. Only four patients < 31 years died during OAT; all four died of overdose

^b^One patient with non-organic psychosis (F29) as the cause of death was included in the group “Other/unknown cause of death”

Cancer and cardio-vascular and pulmonary diseases were the most frequent somatic causes of death. Twenty-six patients died of cancer, and lung cancer alone accounted for one-third of cancer fatalities. COPD, emphysema and pneumonia were the most frequent causes of death for those who died of pulmonary diseases. Cardio-vascular causes of death were more diverse, involving pulmonary embolism, haemorrhagic stroke, endocarditis, chronic ischaemia or myocardial infarction. Among the 14 patients who died of a liver disease, one died of liver cancer. The group “Other somatic cause of death” included four cases of kidney failure, three of diabetes, two of gastrointestinal bleeding, two of bacterial infections/sepsis and one case of epilepsy. Seven patients had a confirmed secondary amyloidosis (amyloid A [AA] amyloidosis) diagnosis with end-stage kidney disease and needed regular haemodialysis, but only two of them had kidney failure as the underlying cause of death. Bacterial infections contributed substantially to mortality: 30 patients (15%) had bacterial infections either as a contributing cause or as an underlying cause of death in the Cause of Death Registry. The most common infections were pneumonia, endocarditis or fatal sepsis. Nine patients (5%) had human immunodeficiency virus (HIV), but no patients died of acquired immune deficiency syndrome (AIDS).

Several patients had more than one potential fatal somatic disease documented in medical records or in the Cause of Death Registry. Two fatalities exemplified the complex of multiple comorbidities: one involved chronic hepatitis B and C, AA amyloidosis with end-stage kidney failure, COPD and death due to overdose; the other involved chronic hepatitis B and C, HIV, COPD, acute liver and kidney failure and death due to respiratory failure.

Among the 84 drug-induced deaths, 71 patients had undergone an autopsy. In the Cause of Death Registry, methadone was reported as the main intoxicant in 31 deaths and heroin in 17. Other opioids, including buprenorphine, were the reported main intoxicant in an additional 15 deaths. No drug-induced deaths occurred in the first month after initiation of methadone or buprenorphine. Ten of the 17 patients with substance use disorder as an underlying cause of death had severe medical comorbidities as a contributing cause of death in the Cause of Death Registry.

Half of the violent deaths were suicides, and three-quarters of the suicides were intentional self-harm by hanging. Both men and women died in suicides and accidents (falling, hypothermia, fire and traffic accidents), but all three homicide victims were men.

Forensic or medical autopsies were performed for 125 (63%) of the deaths. The autopsy rate was high for all unnatural deaths: 66% for suicides, 85% for drug-induced deaths, 88% for accidents and 100% for homicides. The most common place of death was the home address (43%), where almost two-thirds of the deaths were drug-induced; 37% died in a hospital or other health institution, three-quarters of whom died of an already known somatic disease. We found no statistically significant differences between men and women in the causes of death, autopsy rates or place of death.

### CMRs

Table 3 shows that the mean number of patients in OAT was 7220 in 2014 and 7439 in 2015, giving a total observation period of 14,659 PY. The 2-year all-cause CMR during OAT was 1.4/100 PY (equivalent to 1.4%). In general, CMRs increased with age. The mortality rate for somatic causes of death was twice as high in patients aged > 50 years than in those aged 41–50 (mortality rate ratio [MRR] 2.1, CI 1.3–3.4). The rates for drug-induced deaths also increased with age, although not as steeply as those for somatic causes of death, whereas the rates for violent deaths were the same across all age groups. Men had a slightly higher mortality rate than women (MRR 1.2, CI 0.5–0.9). The mortality rate was twice as high among patients taking methadone than among those taking buprenorphine (MRR 2.0, CI 1.5–2.7).

**Table 3:** CMRs/100 PY (95% CI) during OAT, stratified by age, OAT medication and gender

	PY in OAT (%)	Deaths, n (%)	CMR/100 PY (95% CI)	Drug-induced cause of death	Somatic cause of death	Violent cause of death
2014	7220	95 (48)	1.3 (1.1–1.6)	NA	NA	NA
2015	7439	105 (52)	1.4 (1.2–1.7)	NA	NA	NA
*Total*	14,659	200	1.4 (1.2–1.6)	0.6 (0.5–0.7)	0.6 (0.5–0.8)	0.2 (0.1–0.2)
*Age*						
< 41 years	5570 (38)	33 (17)	0.6 (0.4–0.8)	0.3 (0.2–0.5)	0.1 (0.1–0.2)	0.2 (0.1–0.3)
41–50 years	5424 (37)	81 (41)	1.5 (1.2–1.9)	0.7 (0.5–0.9)	0.7 (0.5–0.9)	0.2 (0.1–0.3)
> 50 years	3665 (25)	86 (43)	2.4 (1.9–2.9)	0.8 (0.6–1.2)	1.4 (1.0–1.8)	0.1 (0.0–0.3)
*OAT medication*						
Methadone	5707 (39)	109 (55)	1.9 (1.6–2.3)	0.8 (0.6–1.1)	0.9 (0.7–1.2)	0.2 (0.1–0.4)
Buprenorphine	8487 (58)	82 (41)	1.0 (0.8–1.2)	0.4 (0.3–0.6)	0.4 (0.3–0.6)	0.1 (0.1–0.2)
*Gender*						
Male	10,261 (70)	147 (73)	1.4 (1.2–1.7)	0.6 (0.4–0.7)	0.7 (0.5–0.9)	0.2 (0.1–0.3)
Female	4398 (30)	53 (27)	1.2 (0.9–1.6)	0.6 (0.4–0.8)	0.5 (0.3–0.7)	0.2 (0.1–0.3)

*CMR* crude mortality rate, *PY* person-years, *CI* confidence interval, *OAT* opioid agonist treatment, *NA* not applicable

Causes of death *n* = 197, three patients with other/unknown cause of death excluded

OAT medication *n* = 191, other OAT medication excluded. This represents the use of OAT medication at the time of death. We could not obtain information about the changes in OAT medication before death

### Characteristics associated with drug-induced cause of death during OAT

Table 4 shows the results from a multilevel logistic regression analysis assessing characteristics associated with drug-induced cause of death compared with all other causes of death during OAT. In bivariate analyses, both increasing age (*p* < 0.05) and increasing Charlson comorbidity index score (*p* < 0.001) were associated with lower odds of dying of a drug-induced cause of death. In the multiple model, only the Charlson comorbidity index remained significant (p < 0.001). The variables of male gender, taking methadone (compared with taking buprenorphine), previous non-fatal overdoses, psychiatric admissions and duration of OAT were not associated with dying of a drug-induced cause of death during OAT, neither in the bivariate nor in the multiple analyses.

**Table 4:** Results of multilevel logistic regression analysis for characteristics associated with drug-induced cause of death during OAT^a^

Characteristics	Bivariate models	Multiple model
	OR (95% CI)	OR (95% CI)
Gender		
Men	1	1
Women	1.37 (0.70; 2.70)	1.59 (0.77; 3.30)
Age	0.95 (0.92; 0.99)*	0.99 (0.95; 1.04)
OAT medication		
Buprenorphine	1	1
Methadone	1.24 (0.64; 2.41)	1.25 (0.63; 2.48)
Charlson index	0.73 (0.62; 0.85)**	0.72 (0.61; 0.86)**
Non-fatal overdoses^b^		
No	1	1
Yes	1.60 (0.83; 3.10)	1.72 (0.82; 3.60)
Psychiatric admissions^c^		
No	1	1
Yes	1.35 (0.70; 2.60)	0.91 (0.44; 1.88)
OAT total duration in years	0.97 (0.90; 1.04)	1.00 (0.92; 1.08)

*OAT* opioid agonist treatment. Only complete cases are included, *N* = 181. *p < 0.05, **p < 0.001

^a^The reference category is “Other causes of death”

^b^Non-fatal overdoses registered in the NPR or in OAT status reports in the last 5 years before death

^c^Psychiatric admissions registered in the NPR in the last 5 years before death

## Discussion

In this study on mortality in the total Norwegian OAT population, both somatic and drug-induced causes of death were frequent during OAT. In the 2-year observation period, 1.4% of the patients died. In general, CMRs increased with age, and this pattern was more pronounced for somatic causes than other causes of death. The CMR was also higher in patients taking methadone compared with buprenorphine. In the multiple regression model, we found that increasing somatic comorbidity, as measured by the Charlson comorbidity index, reduced the odds of dying of a drug-induced cause of death compared with other causes of death.

In line with previous Norwegian studies, we found that somatic causes of death among OAT patients predominated [8, 17]. Non-communicable diseases such as cancer and COPD take time to develop and are associated with both age and the lifestyle factors prevalent among OAT patients. High rates of pulmonary diseases and increased cancer risk are consistent with previous findings in ageing OAT patients [27–31]. COPD and emphysema are independent risk factors for lung cancers, together with smoking, and predict reduced survival [32, 33]. Liver cirrhosis and liver cancer due to hepatitis C also contribute substantially to morbidity and mortality among opioid users [34]; however, despite a high prevalence of hepatitis C among the deceased in our study, only 14 patients died of liver disease. Some of the deaths of somatic origin were probably more directly associated with injecting drug use. Acute bacterial skin and soft tissue infections are common among injecting drug users [35], and bacteraemia often causes severe focal infections and sepsis. Persisting infections and inflammation caused by continued injecting and skin popping (subcutaneous injecting) are also associated with AA amyloidosis [36, 37]. AA amyloidosis was not encountered among heroin users in Norway until 2005 [37] but is now an emerging issue among the ageing OAT patients. Injection-related health risks other than blood-borne viral infections in OAT patients who continue to use drugs might be an under-researched topic.

Although the overdose risk is reduced during OAT, nevertheless 42% of the patients in our study had a drug-induced cause of death. None of the patients died of an overdose in the first month after initiating OAT. The increased risk of fatal overdose during initiation of methadone may vary according to treatment setting [4, 38]. According to Norwegian OAT guidelines, both buprenorphine and methadone should be initiated under monitoring and observation, and inpatient detoxification at the initiation of OAT is common [19]. Methadone was judged to be the main intoxicant in 31 of the 84 drug-induced deaths and, in all except two cases, the patient was taking methadone as the OAT medication. The interpretation of this finding is not straightforward. It is difficult to determine the precise role of OAT medication in fatal overdoses [39]. The instituted dose of methadone may become dangerous because of increasing vulnerability as OAT patients age and comorbidity levels rise. The overdose risk among OAT patients is associated with several factors such as somatic and psychiatric comorbidities, co-prescribing, previous non-fatal overdoses and polydrug use [40–42], which may make it difficult to ascertain the exact cause of death. In addition, the post-mortem examiner is not always informed about the OAT status. Thus, the number of methadone deaths might represent an overestimation, and may in fact have been caused by single or multiple somatic causes in combination with regular prescribed methadone doses.

An all-cause mortality rate of 1.4/100 PY during OAT was the same as found in an earlier Norwegian study [17], but higher than the rate of 0.93/100 PY found in a systematic review and meta-analysis [4]. In line with previous studies, CMRs increased with age, and were higher in men and for patients taking methadone compared with buprenorphine [2–4, 8]. Suggested explanations for increased CMRs among patients taking methadone are methadone-induced prolongation of the QTc interval, increasing the risk of ventricular cardiac arrhythmia (torsades de pointes) and “sudden death”, ingestion of alcohol and BZD, physical comorbidities and harder-to-support patients [15, 16]. In the Norwegian setting, the difference in CMRs might be explained by a “veteran effect”. Until 2001, methadone was the only OAT medication. Patients taking methadone in our study had been treated in OAT for significantly longer than those taking buprenorphine, and most likely had a longer drug career. In addition, patients with a severe or terminal disease such as cancer taking buprenorphine are often converted to methadone or other opioids.

In the regression analysis, we found an association between increased somatic morbidity and reduced odds of a drug-induced cause of death. The Charlson comorbidity index was moderately correlated with age, which could be one explanation why age did not remain significant in the multiple model. Multimorbidity (i.e., having two or more chronic diseases) is associated with increased risk of mortality, functional decline, polypharmacy, increased number of hospital admissions and poorer quality of life [43]. Multimorbidity usually increases with age [43], but patients in OAT have high rates of chronic diseases across all age groups [44, 45]. Several of the patients in our study had multiple severe and potentially fatal medical conditions, and thus several competing disease end-points.

Somewhat surprisingly, given the superior safety profile of buprenorphine, we did not find that taking methadone increased the odds of drug-induced cause of death compared with buprenorphine. The lack of association between the other covariates and drug-induced cause of death could be because the two groups were quite similar, which makes differences less likely to detect. Risk factors not included in the model (e.g., prescription medication, drug use) could be another explanation.

Our findings have several implications. Multimorbidity in OAT patients calls for a broad range of patient-oriented and organizational measures, such as improved treatment and follow-up of chronic diseases and multidisciplinary teamwork and co-ordination of care [43, 44]. The high prevalence of COPD and pulmonary cancer suggests that a stronger focus on tailored tobacco harm-reduction approaches and smoking cessation is important for this patient group, and as early in their lives as possible, to reduce cumulative risk. OAT patients should be offered spirometry and lung image tests [32, 33]. Overdose prevention is a multifaceted challenge [14]. Further measures may include improved follow-up after non-fatal overdose, reviewing older patients’ methadone dosage in the context of somatic comorbidities (e.g., reduced liver and kidney function) and offering regular electrocardiograms to patients aged > 45 years. Distribution of intranasal naloxone to at-risk populations is also relevant [15, 46, 47].

### Strengths and limitations

The strengths of our study include the use of register data that were combined with information from hospital medical records. This gave in-depth information about the fatalities that were not accessible using register data alone. The national OAT programme is organized within the public specialist health care service in Norway, and has a monopoly of this treatment modality; thus, we were able to study mortality in a complete, national OAT population. The high rate of forensic or medical autopsy also strengthens the validity of the findings. A valid cause of death was not established in only two patients (1%).

Our study has several limitations. Almost half (47%) of the questionnaires were completed by clinicians other than physicians, who do not always have access to somatic medical records. Thus, we cannot rule out the possibility of information bias. Regarding somatic comorbidity, we have no data on smoking status, but the smoking prevalence among Norwegian OAT patients is high and similar to the 69–94% prevalence reported in earlier studies [27, 45, 48, 49]. In addition, the number of non-fatal overdoses is probably under-estimated, because most overdoses in Norway are attended by the ambulance service only. A higher number of participants would have allowed for more variables in the regression analysis. We did not have information on the changes in variables that can vary over time, such as prescription of BZD, psychotropic medications and changes in OAT medication before death. The broad categories of prescribed medication (at least one prescription of benzodiazepine and psychotropic medication in the year before death) limited their use as covariates in the regression analyses.

## Conclusions

In this study on mortality among patients in the Norwegian OAT programme, both somatic and drug-induced causes of death were common during OAT. AA amyloidosis is an emerging issue. As expected, CMRs increased with age, and this increase was steeper for somatic causes than for other causes of death. CMRs were also higher in men and in patients taking methadone. Increasing somatic comorbidity reduced the odds of a drug-induced cause of death. Both improved treatment and follow-up of chronic diseases, especially in patients aged > 40 years, and continuous measures to reduce drug-induced deaths appear to be essential to reduce future morbidity and mortality burdens in this population.

## Data Availability

The dataset generated and analysed during the current study is not publicly available to protect the privacy of participants but it is available from the corresponding author on reasonable request.

## Abbreviations

AA: Amyloid A
AIDS: Acquired immune deficiency syndrome
BZD: Benzodiazepines
CI: Confidence interval
CMR: Crude mortality rate
COPD: Chronic obstructive respiratory disease
GP: General Practitioner
HIV: Human immune deficiency virus
ICD-10: International Classification of Diseases, 10th edition
MRR: Mortality rate ratio
NPR: Norwegian Patient Registry
OAT: Opioid agonist treatment
OR: Odds ratio
OUD: Opioid use disorder
PY: Person-years
SD: Standard deviation
